# The impact of capitalist profit-seeking behavior by online food delivery platforms on food safety risks and government regulation strategies

**DOI:** 10.1057/s41599-023-01618-w

**Published:** 2023-03-25

**Authors:** Xiaoting Dai, Linhai Wu

**Affiliations:** 1 School of Business, Jiangnan University, Wuxi, China; 2 Institute for Food Safety Risk Management, Jiangnan University, Wuxi, China

**Keywords:** Social policy, Economics, Environmental studies

## Abstract

Capital monopolized platforms that have emerged based on the new “Internet Plus” economic form will undoubtedly distort market competition. Taking the Meituan online food delivery platform in China as an example, this study (1) investigates the game of interests between the platform and restaurants and the resulting impact on food safety risks, and (2) explores the interactions among government regulations, platform profit strategy, and restaurant behavior. An evolutionary game model between the capital-monopolized online food delivery platform and the restaurants was developed with an optional promotion fee and government regulation level as variables. Analysis of four equilibrium situations derived from the evolutionary game model showed that the platform always pursued high overall profit in every equilibrium situation. This capitalist profit-seeking behavior will most likely reduce the profit margins and even the survival space of restaurants on the platform, forcing them to engage in opportunistic behavior and illegal production, thereby resulting in increased food safety risks in online food delivery and consequently increased government regulation costs. Although increased government regulation can change the production strategy of restaurants, it cannot change the platform’s capitalist profit-seeking behavior. The platform’s overall payoff is not reduced due to increased regulation, which once again proves the profit-seeking nature of capital. The strategy of low commissions but high promotion fees may require greater government regulation to restrain the opportunistic behavior of restaurants. Therefore, the Chinese government regulators can achieve a win-win situation of improved government regulation efficiency and reduced regulation costs by designing new regulatory strategies that do not reduce the platform’s overall payoff.

## Introduction

In the context of the rapid development of a new economic form called “Internet Plus”, the platform economy, which is a new economic system based on a series of digital technologies driven by the Internet (Xue et al., 2020), has rapidly developed around the world. In particular, the platform economy has greatly invigorated the food consumption market, especially the restaurant industry, and yielded a new form of food consumption, namely the online food delivery industry (Yeo et al., 2017; Kassi and Lehdonvirta, 2018; Cho et al., 2019; Gunden et al., 2020; Cheng et al., 2021). Online food delivery has gradually become a daily necessity, especially during the prevention and control of the COVID-19 pandemic (Oncini et al., 2020; Li et al., 2022; Lu et al., 2022), and gave birth to the rapid development of online food delivery platforms (hereinafter referred to as the “platform”) around the world. Typical examples are “Uber Eats” in the United States, “Deliveroo” in the United Kingdom, “Swiggy” in India, “iFood” in Brazil, and “Ele.me” and “Meituan” in China (Li et al., 2020). From 2019 to 2021, the total market size of China’s restaurant industry did not change significantly, but the market size of online food delivery increased by 61.62%. In 2021, there were 544 million online food delivery users in China (CNNIC, 2022). Moreover, due to China’s huge population, the online food delivery industry is developing more rapidly in China than elsewhere, with the penetration rate 1increasing from 3.87% in 2015 to 19.92% in 2021 (Huaan Securities, 2021; China Hotel Association et al., 2022).

However, although online food delivery services provide convenience for consumers, they may also increase food safety risks. Compared with traditional food consumption where food is prepared and consumed on the restaurant’s premises, online food delivery involves some unique features, such as ordering on third-party platforms, food being handled in packaging, and replying to individuals for distribution, all of which provide a window for secondary pollution. Moreover, online platforms directly connect scattered edible agricultural products with food producers, traders, and widely dispersed consumers, forming a direct connection between food producers/traders and consumers, which introduces new difficulties into the existing government food safety regulation system. Furthermore, online platforms enjoy a unique monopoly advantage by acquiring data on most online food retailers and consumers through big data analysis (Saad, 2020). The platforms may affect the normal operation of the online food delivery market by means of digital information technology. For example, the platforms provide additional compensation to specific consumers in the form of virtual coupons in exchange for the consumers deleting complaints and negative comments. More seriously, the platforms may hinder the effective flow of real food safety information between the platforms and government regulators. There is high information asymmetry among platforms, food producers and traders, consumers, and the government (Du et al., 2019), which makes government regulation more difficult. Thus, all these factors mean that great safety risks exist in online food delivery (Zhang, 2021).

Online platforms are one of the emerging service industries that have attracted capital in recent years. In pursuit of high returns, massive capital investment or even disorderly expansion will inevitably lead to the emergence of industry leaders or large monopolistic interest groups, which distort market behavior and affect the orderly operation of the platform economy (Pigatto et al., 2017). This has been demonstrated in real estate, finance, education, healthcare, entertainment, and other fields (Fibozzi and Xiao, 2019; Robison et al., 2020; Jarrow and Kwok, 2021). In recent years, capital-controlled monopolies have also emerged in the online food delivery industry. The capitalist profit-seeking instinct only considers market share, financial returns, expanding network externalities, and increasing the platform capacity of restaurants and consumers, while ignoring food safety and other social responsibility issues. For example, the insufficient qualification review carried out by platforms before enrolling restaurants has resulted in the entry of a large number of non-compliant restaurants into the market, thus increasing food safety risks (Cheng and Dong, 2017). The reality in China is that traditional regulatory measures are ineffective when applied to the online food delivery industry due to higher information asymmetry and market fragmentation. As a result, it has become increasingly challenging for the government to efficiently allocate regulatory resources and develop effective regulation strategies. Moreover, different from traditional industries, the online food delivery industry requires government intervention to balance the interests of capital-monopolized online food delivery platforms with those of food producers/traders (i.e., restaurants) to prevent the increase in food safety risks as a result of the game between them.

The online food delivery industry in China is characterized by rapid development, a large number of users, a large scale, and a high penetration rate. According to Statista (2022), the online food delivery industry in China is forecast to earn revenues of US$158.1 billion in 2022, compared with about US$66.5 billion in the United States. Due to the uncertainty of the COVID-19 pandemic, and more importantly, the convenience and relatively low prices of food ordered online, it is foreseeable that the online food delivery industry will develop faster in China in the future, a uniqueness that other countries may not have. Accordingly, this study aims to (1) develop an evolutionary game model between delivery platforms and restaurants in the capital-monopolized online food delivery market in order to explore the impact of the commission and promotion fee strategies implemented by these platforms on the production strategies of restaurants, as well as the resulting impact on the food safety risks in online food delivery, and (2) investigate the interactions among government regulation, platform profit strategy, and restaurant behavior. Furthermore, this study validates the conclusions drawn from the evolutionary game model based on the online food delivery market data of China and explores different government regulatory strategies. Although China’s social system is quite different from that of Western countries, capital engages in profit-seeking behavior no matter where in the world it exists (Keith,^1967^; Pigatto et al., 2017). Hence, online food delivery platforms around the world have similar market characteristics (Fibozzi and Xiao, 2019; Robison et al., 2020; Jarrow and Kwok, 2021), and governments face similar challenges in enacting regulations. Therefore, the conclusions of this study undoubtedly have universal implications.

## Literature review

The platform is a product of the development of the digital economy. Platforms are the face of a new economic development model that places data control at their core, enables precise matching and consumption distribution through statistical analysis of information, and connects multiple markets to promote transactions. Studies have shown that the operating mode of the platform economy not only caters to consumers’ needs for diversification, personalization, and convenience, reduces operating costs, expands the scope of business, and improves the efficiency of bilateral user transactions, thereby achieving win-win cooperation, but it also broadens the marketing channels of restaurants and promotes the development of trading platforms, logistics and express delivery, packaging materials, and other related industries (Evans, 2003; Hagiu, 2014; Gawer, 2014; Langley and Leyshon, 2017; Kenny and Zysman, 2020; Hill, 2021; Yao and Xu, 2021). In online transactions that rely on information transmission and connect highly fragmented small and micro enterprises and consumers, because the information obtained by consumers is separated from food entities and the physical space of transaction parties, these transactions inevitably involve greater information asymmetry than those carried out within traditional industries. Moreover, when the online platform uses information technology as a control tool for economic behavior, food quality cannot be guaranteed, and low-quality products masquerading as high-quality ones will eventually emerge (Wei and Yao, 2020). Srnicek (2017) suggested that advanced capitalism in the early 21st century took advantage of platform data to create high information asymmetry to achieve monopolies and high returns.

Food is a commodity, but it also has special attributes that differ from those of ordinary commodities, and it has some characteristics of public goods because it is related to physical health (Wu et al., 2020; Xiao and Yang, 2022). Moreover, food has the attributes of search, experience, and credence goods (Casewell and Padberg, 1992). The credence attributes of food make it difficult for consumers to identify safety information even after a purchase (McCluskey, 2000; Grunert, 2002), and an unavoidable information asymmetry exists between consumers and producers (Ortega et al., 2011). However, online food delivery not only involves the separation of transaction parties in physical space, but also the separation between the food consumers want to buy and the information they have, which leads to more serious information asymmetry and further highlights the credence attributes.

Moreover, in online transactions based on information technology, the platforms or restaurants control consumers and act to promote their own financial interests by means of digital information technology and complete data, making it difficult to guarantee food quality, and resulting in a ‘market for lemons’ (Wei and Yao, 2020). In this case, it is necessary for a regulator (government) that is trustworthy enough among consumers to intervene in and regulate the market in accordance with laws and regulations to ensure food safety. Therefore, government regulation of food sold through online platforms is quite different from that of other commodities, such as clothing, sold online. For example, regulation of clothing sold online requires random inspection of product identification and fiber content labeling. However, this is not sufficient for the regulation of food sold through online food delivery services, for which real-time regulation is more effective.

Platforms continue to achieve economies of scale through acquisitions and in turn gain more data and profits by virtue of their dominant market position (Rossotto et al., 2018). Couldry and Mejias (2018) argue that large technology platforms will employ quantitative calculations to squeeze others after the concentration of power in their hands. Chen et al. (2022) and Van Veldhhoven et al. (2021) argue that online food delivery platforms do not necessarily help increase the total demand for restaurants and that paying commissions to platforms for the purpose of attracting customers may ultimately damage restaurants’ profitability; moreover, the platforms may also impose more unreasonable costs on small and micro enterprises, for example, by forcing them to bear high responsibility for refunds.

As a result, many restaurants opt to reduce their brick-and-mortar area to save costs, which ultimately leads to a proliferation of so-called ghost kitchens^2^ (Nita, 2019; Belleri, 2020). Li et al. (2020) believe that the food safety risks and consumer health problems caused by ghost kitchens are high and thus pose a challenge to the effectiveness of government regulation. Meanwhile, in order to survive or earn higher profits, restaurants may engage in opportunistic behavior^3^ that deceives consumers (Wadleigh et al., 2015; Rees, 2020, Wei and Yao, 2020; Rezazade et al., 2022).

At present, although there is an overall trend of improvement in food safety in China, the situation is still complex and severe, especially in production and processing, where opportunistic behaviors such as counterfeiting and fraud have not been effectively curbed (Wu et al., 2021). Barinda and Ayuningtyas (2022) believe that sharing regulatory resources, maintaining a balance between stakeholders, and continuous updating of scientific knowledge are all important factors in designing a food safety risk control system. Online food delivery platforms, which directly connect food producers/traders and consumers by leveraging Internet technology, have only begun to enter the public eye in the last 10 years (Zhao et al., 2021). Because these platforms directly reach the dining table, they circumvent the traditional regulation model, which has failed to keep pace with these new developments in the market and has thus been rendered ineffective (Zhao, 2017; Wu et al., 2019). Although Chinese government regulators are accelerating the development of “Internet Plus smart regulation” technology, it still lags behind the Internet information technology that supports the rapid development of the platforms. Capital monopolized platforms affect fair competition in the restaurant market, which not only makes real-time monitoring difficult for the government but also increases information asymmetry and food safety risks in online food delivery (Zhang, 2021; Wang, 2021).

In response to these regulatory difficulties, studies have been conducted on strategies to regulate food safety risks in online food delivery. Cheng and Dong (2017) suggest the importance and necessity of self-regulation by online food ordering platforms. However, Wei et al. (2017) believe that the level of supervision authorized by the government over the platform is sensitive and that if platforms are the sole regulator of the market, their self-interests will drive them to over-expand, overly emphasize their regulatory effectiveness, and exaggerate their rights, which will lead to inadequate regulation of illegal restaurants, e.g., restaurants engaged in counterfeiting and fraud. Meanwhile, other studies have described a new food safety regulation model that combines information sharing, government intervention, and market mechanisms, thereby providing clear guidance on regulation strategies (Yin et al., 2020; Wang et al., 2020).

To sum up, previous research on the online food delivery industry is limited to descriptive research and has mostly been based on conceptual or theoretical elaboration and qualitative analysis. However, there is little research based on quantitative analysis or mathematical modeling. In terms of research content, previous research has not discussed the food safety issue, which is consumers’ most important concern about online food delivery, nor have studies examined strategies to reduce food safety risks in online food delivery from the perspectives of capitalist profit-seeking, the game of interests between the platform and restaurants, and changes in government regulation of the platforms. To this end, this study takes Meituan, an online food delivery platform in China, as an example, and tries to analyze the impact of the platform’s capitalist profit-seeking business model centered on the pursuit of high overall profits on the production strategy of restaurants and food safety risks in online food delivery; furthermore, this study explores the effectiveness of government regulation strategies in different situations.

## Construction and analysis of evolutionary game model

### Background on principal–agent theory

According to the principal–agent theory, the party with information superiority in the transaction is the agent, the inferior party is the principal, and the interest relationship between them is the principal–agent relationship. Both the principal and the agent are rational economic agents who pursue the maximization of their own interests. Inconsistent preferences can easily induce the agent to act against the interests of the principal, which is called the principal–agent problem (Sappington, 1991). Analyzing the complex transactions of online food delivery platforms under this framework reveals a four-tier principal–agent relationship (Jian et al., 2014; Wang and Zhang, 2017): The first tier is the principal–agent relationship between consumers and the platform. Consumers register as buyers on the platform, appoint the platform to match transaction information and establish transactional contracts, and must abide by the rules of the platform, which constitutes the principal–agent relationship between consumers and the platform, with the consumers being the principal, and the platform being the agent. The second tier is the principal–agent relationship between the platform and included restaurants. Restaurants register as sellers on the platform. The platform appoints the restaurants to perform transactional contracts on the basis that they abide by the rules of the platform, which constitutes the principal–agent relationship between the platform and the restaurants. The third tier is the principal–agent relationship between consumers and restaurants. Consumers appoint restaurants to provide food through the platform and provide reasonable prices, repeated purchases, and word-of-mouth communication as incentives to implement participation constraints. The fourth tier is the principal–agent relationship between the government regulator (referred to as the government) and the platform. The principal is the government, and the agent is the platform. The government is responsible for regulating the online transactions of the platform and its restaurants in accordance with current regulations.

**Fig. 1:**
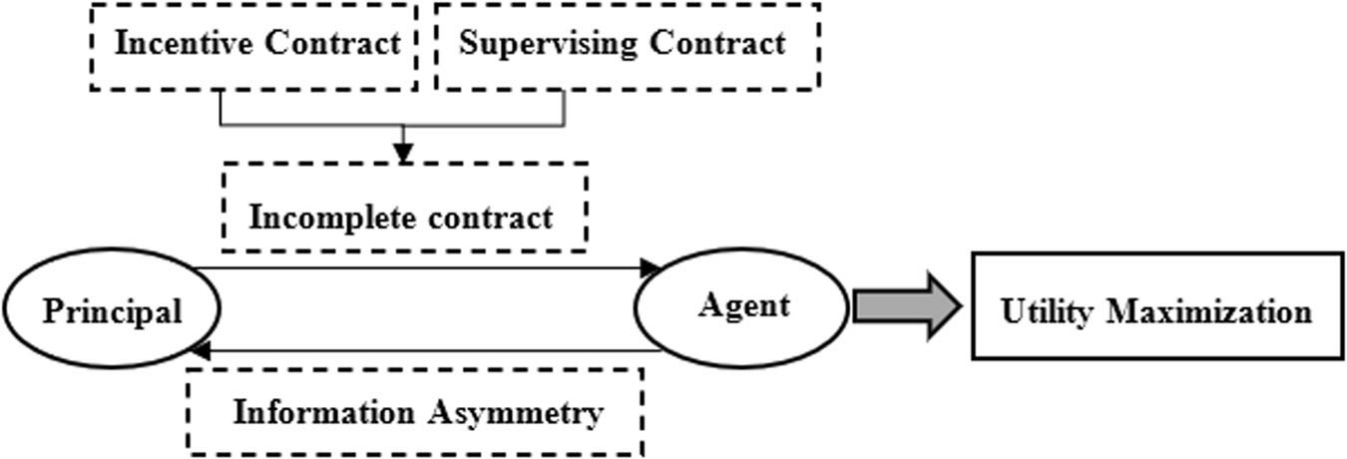
Schematic diagram of principal–agent relationship. In one principal–agent relationship with incomplete regulation and incentive contracts, agents with information and resource advantages will maximize their utility through the incomplete contract space.

In this four-tier principal–agent relationship with incomplete regulation and incentive contracts, agents with information and resource advantages will maximize their utility through the incomplete contract space (Fig. 1), resulting in moral hazard and adverse selection. For example, in the second tier, the platform cannot observe the efforts of restaurants due to the information asymmetry between the platform and restaurants, thus leading to frequent food safety problems. Furthermore, the market may be eventually occupied by non-compliant restaurants, with compliant restaurants squeezed out of the market, thereby increasing food safety risks. In this case, the platform can restrict the behavior of restaurants by using incentives and punishments. Hence, the platform’s regulation strategy is particularly important. The platform should implement regulation strategies such as setting strict requirements for entry of restaurants, intervening in the operations of restaurants, increasing the frequency of random inspections, and strengthening punishments.

The problem of online food delivery platforms discussed in this study involves the aforementioned four-tier principal–agent relationship among the government, the platform, restaurants, and consumers. The transmission of interests among the four actors involves complicated mechanisms. Moreover, online transactions have an open transaction environment, virtual transaction parties, concealed transaction behavior, and various transaction methods. To this end, this study develops an evolutionary game model between the capital-monopolized online food delivery platform and restaurants, and investigates the game of interests between the platform and restaurants and the resulting impact on food safety risks in online food delivery from the perspective of dynamic evolution, in order to provide guidance for the government to develop regulatory policies based on the game balance results and reality.

### Hypotheses of the platform–restaurant game model

Based on the current state of the online food delivery market in China, this paper constructs an evolutionary game model of interests between online food delivery platform and restaurants. Generally speaking, the online food delivery operation model in China is one where food is produced by restaurants and sold to consumers through the ordering platform, and the platform charges a percentage of the sales according to the contract with the restaurants. The platform does not produce food, and they receive money for each sale. Meanwhile, the platform provides restaurants with optional paid promotion services aiming to increase the exposure of restaurants in the consumer market to increase their market share. Government regulators (referred to as the government) regulate the online food delivery industry in accordance with the principle of localization. The model hypotheses are as follows:

Hypothesis 1: Major players in the model are the online food delivery platform (referred to as the platform) and restaurants on the platform. The set of strategies adopted by the platform is {low commission, high commission}, and the corresponding probabilities are *xx* and 1−*x*_1_−*x*, respectively; the set of strategies adopted by merchants in order to gain profits is {safe production, illegal production}, and the corresponding probabilities are *yy* and 1−*y*_1_−*y*, respectively; and 0 ≤ *x* ≤ 10 ≤ *x* ≤ 1, 0 ≤ *y* ≤ 10 ≤ *y* ≤ 1.

Hypothesis 2: *ββ* is the commission rate charged by the platform to the restaurants, and *MM* is the promotion fee. *S*_0_ *S*_0_ is the revenue earned by restaurants selling food without any promotion, and the revenue of the platform is *βS*_0_ *βS*_0_ in this case. *S*_1_ *S*_1_ is the revenue of restaurants selling food using the platform’s promotion services, and platform’s revenue is *βS*_1_ + *MβS*_1_ + *M* in this case. *C*_0_ *C*_0_ is the platform’s daily operating cost, *C*_1_ *C*_1_ is the cost of safe food production for restaurants, and *C*_2_ *C*_2_ is the cost of illegal food production for restaurants (*C*_1_ > *C*_2_ *C*_1_ > *C*_2_).

Hypothesis 3: When the platform implements a low-commission strategy, the probability that restaurants will choose promotion services is *α*_0_ ≤ *α* ≤ 1*α* (0 ≤ *α* ≤ 1); however, when the platform implements a high-commission strategy, restaurants generally do not choose promotion services.

Hypothesis 4: *ωω* is the probability of the government identifying illegal production by restaurants (0 ≤ *ω* ≤ 10≤ *ω* ≤1). Punishment of illegal production incurs greater losses, such as reputation loss and fines, when restaurants are utilizing promotion services. *F*_0_ *F*_0_ and *F*_1_ *F*_1_ are the platform’s losses from government punishment of illegal production of restaurants not receiving and receiving promotion services, respectively (*F*_1_ > *F*_0_ *F*_3_ *F*_2_ *F*_1_ > *F*_0_). *F*_3_ and *F*_2_ are the restaurant’s losses from government punishments of illegal production when not receiving and receiving promotion services, respectively (*F*_2_ > *F*_3_ *F*_2_ > *F*_3_). The evolutionary game payoff matrix of the platform and restaurants are shown in Table 1.

**Table 1:** The evolutionary game payoff matrix of the platform and restaurants.

The platform	The restaurants	
	Safe production (*y*)	Illegal production (1−*y*)
Low commission (*x*)	*α*(*βS*_1_−*C*_0_+*M*)+(1−*α*)(*βS*_0_−*C*_0_); *α*(*S*_1_−*C*_1_−*βS*_1_−*M*)+(1−*α*)(*S*_0_−*C*_1_−*βS*_0_)	*α*(*βS*_1_−*C*_0_+*M*-*ωF*_1_)+(1−*α*)(*β*_*S*0_−*C*_0_−*ωF*_0_); *α*(*S*_1_−*C*_2_−*βS*_1_−*M*−*ωF*_2_)+(1−*α*)(*S*_0_−*C*_2_−*βS*_0_−*ωF*_3_)
High commission (1−*x*)	*βS*_0_−*C*_0_; *S*_0_−*C*_1_−*βS*_0_	*βS*_0_−*C*_0_−*ωF*_0_; *S*_0_−*C*_2_−*βS*_0_−*ωF*_3_

### Evolutionary game analysis between the platform and restaurants

The expected payoffs of the platform when charging low and high commissions are *U*_11_ *U*_11_ and *U*_12_ *U*_12_, respectively, and the average expected return of the platform is *U*_1_ *U*_1_.

1 $$\begin{array}{l}U_{11} = y\left[ {\alpha \left( {\beta S_1 - C_0 + M} \right) + \left( {1 - \alpha } \right)\left( {\beta S_0 - C_0} \right)} \right] + \\ \left( {1 - y} \right)\left[ {\alpha \left( {\beta S_1 - C_0 + M - \omega F_1} \right) + \left( {1 - \alpha } \right)\left( {\beta S_0 - C_0 - \omega F_0} \right)} \right]\\ U_{11} = y\left[ {\alpha \left( {\beta S_1 - C_0 + M} \right) + \left( {1 - \alpha } \right)\left( {\beta S_0 - C_0} \right)} \right] + \left( {1 - y} \right)\\ \left[ {\alpha \left( {\beta S_1 - C_0 + M - \omega F_1} \right) + \left( {1 - \alpha } \right)\left( {\beta S_0 - C_0 - \omega F_0} \right)} \right]\end{array}$$

2 $$\begin{array}{l}U_{12} = y\left( {\beta S_0 - C_0} \right) + \left( {1 - y} \right)\left( {\beta S_0 - C_0 - \omega F_0} \right)U_{12} \\ =y\left( {\beta S_0 - C_0} \right) + \left( {1 - y} \right)\left( {\beta S_0 - C_0 - \omega F_0} \right)\end{array}$$

3 $$U_1 = xU_{11} + \left( {1 - x} \right)U_{12}U_1 = xU_{11} + \left( {1 - x} \right)U_{12}$$

The restaurants’ expected payoffs when engaging in safe or illegal production are *U*_21_ *U*_21_ and *U*_2_ *U*_2_, respectively, and their average expected payoff is *U*_22_ *U*_22_.

4 $$\begin{array}{l}U_{21} = x\left[ {\alpha \left( {S_1 - C_1 - \beta S_1 - M} \right) + \left( {1 - \alpha } \right)\left( {S_0 - C_1 - \beta S_0} \right)} \right]\\ +\, \left( {1 - x} \right)\left( {S_0 - C_1 - \beta S_0} \right) \\ U_{21} = x\left[ {\alpha \left( {S_1 - C_1 - \beta S_1 - M} \right) + \left( {1 - \alpha } \right)\left( {S_0 - C_1 - \beta S_0} \right)} \right] \\+ \,\left( {1 - x} \right)\left( {S_0 - C_1 - \beta S_0} \right)\end{array}$$

5 $$\begin{array}{l}U_{22} = x\left[ \begin{array}{l}\alpha \left( {S_1 - C_2 - \beta S_1 - M - \omega F_2} \right) + \left( {1 - \alpha } \right)\left( {S_0 - C_2 - \beta S_0 - \omega F_3} \right)\end{array} \right]\\ \qquad\quad+\, \left( {1 - x} \right)\left( {S_0 - C_2 - \beta S_0 - \omega F_3} \right)U_{22} \\\qquad= x\left[ {\alpha \left( {S_1 - C_2 - \beta S_1 - M - \omega F_2} \right)+ \left( {1 - \alpha } \right)\left( {S_0 - C_2 - \beta S_0 - \omega F_3} \right)} \right]\\ \qquad\quad+\, \left( {1 - x} \right)\left( {S_0 - C_2 - \beta S_0 - \omega F_3} \right)\end{array}$$

6 $$U_1 = yU_{21} + \left( {1 - y} \right)U_{22}U_1 = yU_{21} + \left( {1 - y} \right)U_{22}$$

The replicated dynamic equations for the platform and restaurants regarding capital profit and restaurants’ choice of production strategy are as follows:

7 $$\begin{array}{l}\frac{{d_x}}{{d_t}} = x\left( {1 - x} \right)\left[ \begin{array}{l}y\left( {\alpha \omega F_1 - \alpha \omega F_0} \right) + \alpha \beta \left( {S_1 - S_0} \right) + \alpha M - \alpha \omega \left( {F_1 - F_0} \right)\end{array} \right] \\ \frac{{d_x}}{{d_t}} = x\left( {1 - x} \right)\left[ \begin{array}{l}y\left( {\alpha \omega F_1 - \alpha \omega F_0} \right) + \alpha \beta \left( {S_1 - S_0} \right) + \ \alpha M - \alpha \omega \left( {F_1 - F_0} \right)\end{array} \right]\end{array}$$

8 $$\begin{array}{l}\frac{{d_y}}{{d_t}} = y\left( {1 - y} \right)\left( {x\left( {\alpha \omega F_2 - \alpha \omega F_3} \right) + C_2 - C_1 + \omega F_3} \right) \\ \frac{{d_y}}{{d_t}} = y\left( {1 - y} \right)\left[ {x\left( {\alpha \omega F_2 - \alpha \omega F_3} \right) + C_2 - C_1 + \omega F_3} \right]\end{array}$$

Let $\frac{{d_x}}{{d_t}} = 0\frac{{d_x}}{{d_t}} = 0$ and $\frac{{d_y}}{{d_t}} = 0\frac{{d_y}}{{d_t}} = 0$, and solve the above replicated dynamic equations. Five equilibrium situations of the evolutionary dynamic system of platform capital profit and restaurant production strategy can be obtained, which are (0, 0), (0, 1), (1, 0), (1, 1), and (*x*_0_ *x*_0_, *y*_0_ *y*_0_), where $x_0 = \frac{{C_1 - C_2 - \omega F_3}}{{\alpha \omega \left( {F_2 - F_3} \right)}}x_0 = \frac{{C_1 - C_2 - \omega F_3}}{{\alpha \omega \left( {F_2 - F_3} \right)}}$ and $y_0 = \frac{{\alpha \omega \left( {F_1 - F_0} \right) - \alpha \beta \left( {S_1 - S_0} \right) - \alpha M}}{{\alpha \omega \left( {F_1 - F_0} \right)}}y_0 = \frac{{\alpha \omega \left( {F_1 - F_0} \right) - \alpha \beta \left( {S_1 - S_0} \right) - \alpha M}}{{\alpha \omega \left( {F_1 - F_0} \right)}}$. According to Friedman (1991), the following Jacobian matrix is obtained:

9 $$A = \left( {\begin{array}{*{20}{c}} a & b \\ c & d \end{array}} \right)A = \left( {\begin{array}{*{20}{c}} a & b \\ c & d \end{array}} \right)$$

where $$a = (1 - 2x)\left[ {y\left( {\alpha \omega F_1 - \alpha \omega F_0} \right) + \alpha \beta \left( {S_1 - S_0} \right) + \alpha M - \alpha \omega \left( {F_1 - F_0} \right)} \right]a = \left( {1 - 2x} \right)\left[ {y\left( {\alpha \omega F_1 - \alpha \omega F_0} \right) + \alpha \beta \left( {S_1 - S_0} \right) + \alpha M - \alpha \omega \left( {F_1 - F_0} \right)} \right]$$, $$b = x\left( {1 - x} \right)\left( {\alpha \omega F_1 - \alpha \omega F_0} \right)b = x\left( {1 - x} \right)\left( {\alpha \omega F_1 - \alpha \omega F_0} \right)$$, $$c = y\left( {1 - y} \right)\left( {\alpha \omega F_2 - \alpha \omega F_3} \right)c = y\left( {1 - y} \right)\left( {\alpha \omega F_2 - \alpha \omega F_3} \right)$$, and $$d = \left( {1 - 2y} \right)\left( {x\left( {\alpha \omega F_2 - \alpha \omega F_3} \right) + C_2 - C_1 + \omega F_3} \right)d = \left( {1 - 2y} \right)\left( {x\left( {\alpha \omega F_2 - \alpha \omega F_3} \right) + C_2 - C_1 + \omega F_3} \right)$$.

If the matrix *AA* satisfies both |*A*| = *ad*−*bc* > 0|*A*| = *ad*−*bc* > 0 and *D* = *a* + *d* < 0*D* = *a* + *d* < 0, the equilibrium point of the replicated dynamic equations is the evolutionary stable strategy (ESS). Table 2 shows the values of *a*, *b*, *c*, *da*, *b*, *c*, and *d* corresponding to the five local equilibrium points of the evolutionary dynamic system of platform capital profitability and restaurant production strategy.

**Table 2:** The values corresponding to the local equilibrium points of the evolutionary game.

Equilibrium Point	*a*	*b*	*c*	*d*
(0, 0)	*αβ*(*S*_1_−*S*_0_) + *α*_*M*_−*α*_*ω*_(*F*_1_−*F*_0_)	0	0	*C*_2_−*C*_1_ + *ωF*_3_
(0, 1)	*αβ*(*S*_1_−*S*_0_) + *αM*	0	0	*C*_1_-*C*_2_-*ωF*_3_
(1, 0)	*α*_*ω*_(*F*_1_−*F*_0_)−*αβ*(*S*_1_−*S*_0_)−*αM*	0	0	*αωF*_2_−*αωF*_3_ + *C*_2_−*C*_1_ + *ωF*_3_
(1, 1)	−*α*_*β*_(*S*_1_−*S*_0_)−*αM*	0	0	*αωF*_3_−*αωF*_2_ + *C*_1_−*C*_2_−*ωF*_3_
(*x*_0,_ *y*_0_)	0	*E*_1_	*E*_2_	0

Obviously, *D* = *a* + *d* = 0*D* = *a* + *d* = 0 at point (*x*_0_ *y*_0_ *x*_0_, *y*_0_) does not meet the ESS conditions. This means that this local equilibrium point does not have an evolutionary stable state and thus is not discussed. The stability analysis of the other four equilibrium points is as follows:

1. When $\frac{{M - \beta \left( {S_0 - S_1} \right)}}{{F_1 - F_0}} <\, \omega\, < \frac{{C_1 - C_2}}{{F_3}}\frac{{M - \beta \left( {S_0 - S_1} \right)}}{{F_1 - F_0}} < \,\omega \,< \frac{{C_1 - C_2}}{{F_3}}$ and $M < \omega \left( {F_1 - F_0} \right) - \beta \left( {S_1 - S_0} \right)M \,< \,\omega \left( {F_1 - F_0} \right) - \beta \left( {S_1 - S_0} \right)$, the only ESS of the system is (0, 0); that is, the platform chooses to charge high commissions to make a profit, and the restaurants engage in illegal production (Table 3).

**Table 3:** System evolutionary stability analysis in Situation (1).

Local equilibrium point	|*A*||*A*|	*DD*	Stability results
(0, 0)	+	−	ESS
(0, 1)	+	+	Unstable point
(1, 0)	Uncertain	Uncertain	Saddle point
(1, 1)	Uncertain	Uncertain	Saddle point
  With moderate government regulation and low optional promotion fees, the platform will charge restaurants high commissions to attain its supreme goal of making high profits. Facing such “exploitation” by the platform, the restaurants will aim to maximize their own interests and, considering the market regulation, will probably take opportunistic behavior, i.e., pursue high profits by engaging in illegal production at the risk of being punished by the government, thereby increasing food safety risks.
2. When $\omega \,>\, \frac{{C_1 - C_2 + \alpha \beta \left( {S_1 - S_0} \right) + \alpha M}}{{F_3}}\omega \,> \,\frac{{C_1 - C_2 + \alpha \beta \left( {S_1 - S_0} \right) + \alpha M}}{{F_3}}$ and $M \,<\, \frac{{C_2 - C_1 + \omega F_3 - \alpha \beta \left( {S_1 - S_0} \right)}}{\alpha }M \,<\, \frac{{C_2 - C_1 + \omega F_3 - \alpha \beta \left( {S_1 - S_0} \right)}}{\alpha }$, the only ESS of the system is (0, 1); that is, the platform chooses to charge high commissions to make profits, and the restaurants engage in safe production (Table 4).

**Table 4:** System evolutionary stability analysis in Situation (2).

Local equilibrium point	|*A*|	*D*	Stability results
(0, 0)	Uncertain	Uncertain	Saddle point
(0, 1)	+	+	ESS
(1, 0)	Uncertain	Uncertain	Saddle point
(1, 1)	Uncertain	Uncertain	Saddle point
  With strong government regulation and low optional promotion fees, the platform will give priority to meeting the short-term interests of shareholders and thus impose high commissions on restaurants. Even if the purpose of maximizing their own interests remains unchanged, considering the strong government regulation and severe potential punishments, restaurants will not choose to take opportunistic behavior and will thus actively engage in safe production because the risk of punishment is greater than the additional payoff from illegal production.
3. When $\omega \,<\, {\rm {min}}\left[ {\frac{{\beta \left( {S_1 - S_0} \right)}}{{F_1 - F_0}},\,\frac{{C_1 - C_2}}{{F_3 + \alpha \left( {F_2 - F_3} \right)}}} \right]\omega \,<\, {\rm {min}}\left[ {\frac{{\beta \left( {S_1 - S_0} \right)}}{{F_1 - F_0}},\,\frac{{C_1 - C_2}}{{F_3 + \alpha \left( {F_2 - F_3} \right)}}} \right]$ and *M* > *ω*(*F*_1_−*F*_0_)−*β*(*S*_1_−*S*_0_)*M* > *ω*(*F*_1_−*F*_0_)−*β*(*S*_1_−*S*_0_), the only ESS of the system is (1, 0); that is, the platform chooses to charge low commissions to make profits, and the restaurants engage in illegal production (Table 5).

**Table 5:** System evolutionary stability analysis in Situation (3).

Local equilibrium point	|*A*|	*D*	Stability results
(0, 0)	Uncertain	Uncertain	Saddle point
(0, 1)	Uncertain	Uncertain	Saddle point
(1, 0)	+	−	ESS
(1, 1)	−	Uncertain	Saddle point
  With weak government regulation and high optional promotion fees, even if the platform’s pursuit of profit remains unchanged, the dual pressure of high commissions and high promotion fees will lead to alienation between the restaurants and the platform and ultimately affect the payoff of the platform, and then the platform may charge low commissions. On the other hand, low *ω* means weak government regulation (i.e., a small financial penalty by the government) that will make restaurants trust their luck, which will increase their probability of engaging opportunistic behavior. Moreover, the platform may choose to turn a blind eye to the illegal production by restaurants to get a greater market share. Facing only insufficient penalties by the government restaurants will probably choose illegal production in order to maximize their own interests. Therefore, in an environment with weak government regulation and high optional promotion fees, the platform may charge low commissions, and the restaurants may choose illegal production.
4. When $\omega \,>\, \frac{{C_1 - C_2}}{{F_3 + \alpha \left( {F_2 - F_3} \right)}}\omega \,>\, \frac{{C_1 - C_2}}{{F_3 + \alpha \left( {F_2 - F_3} \right)}}$ and $M \,>\, \omega \left( {F_1 - F_0} \right) - \beta \left( {S_1 - S_0} \right)M \,>\, \omega \left( {F_1 - F_0} \right) - \beta \left( {S_1 - S_0} \right)$, the only ESS of the system is (1, 1); that is, the platform chooses to earn money by charging lower commissions rather than higher ones, and the restaurants engage in safe production (Table 6).

**Table 6:** System evolutionary stability analysis in Situation (4).

Local equilibrium point	|*A*|	*D*	Stability results
(0, 0)	Uncertain	Uncertain	Saddle point
(0, 1)	Uncertain	Uncertain	Saddle point
(1, 0)	Uncertain	Uncertain	Saddle point
(1, 1)	+	−	ESS

When government regulation is very strong and optional promotion fees are high, as described in Situation (3), the platform will choose to charge low commissions to avoid overly “squeezing” restaurants, which could potentially drive them away from the platform. On the other hand, when government regulation is greatly enhanced, restaurants understand that the risk of punishment is greater than the possible payoff from illegal production, so they will not choose to take opportunistic behavior but instead actively engage in safe production.

Table 7 describes the evolutionary game mechanisms and changing patterns of the platform’s capital profit and restaurants’ production strategy in different situations using the optional promotion fee (*MM*) and government regulation (*ωω*) as variables based on Tables 3–6.

**Table 7:** Summary of evolutionary mechanisms in Situations (1)–(4).

Government regulation	Platform strategy	Restaurant strategy	Corresponding stability
Weak	High promotion fees; Low commissions	Illegal production	(3)
Moderate	Low promotion fees; High commissions	Illegal production	(1)
Strong	Low promotion fees; High commissions	Safe production	(2)
Very strong	High promotion fees; Low commissions	Safe production	(4)

Analysis of Table 7 shows that platform always develops profit-seeking strategies in accordance with the principle of pursuing high overall profits, which confirms the profit-seeking nature of capital. Combining situations (1) and (2) shows that when the platform charges low promotion fees, it will inevitably charge high commissions to ensure its overall profits, and it does not consider whether the restaurants engage in illegal production and thus cause food safety problems.

Furthermore, changes in government regulation levels can change the strategy used by restaurants, but not that of the platform. The platform’s profit-seeking strategy is not affected by changes in government regulations covering restaurants, which fully confirms the profit-seeking nature of capital. Comparing situations (1) and (2) shows that when government regulation shifts from a moderate to a high level, restaurants have a high probability of shifting from illegal to safe production because the probability may increase of a negative payoff due to illegal production being discovered by the government. Even if governments uncover illegal production by restaurants, which causes losses to the platform, the platform will not take the initiative to reduce its overall profits but will still adhere to the strategy of high commissions and low promotion fees.

It is worth noting that the above phenomenon is also seen in situations (3) and (4). The main reason is that in a closed market system, the monopolistic platform has the ability to pass on to restaurants any loss of profits due to increased government regulation and thus can avoid a significant decrease in its own profits. Therefore, government regulation should not focus only on restaurants, but instead, a strategy should be developed to regulate both the restaurants and the platform and focus more on the platform. Only in this way can a balance be better maintained in the game of interests between the platform and restaurants, and the long-term and healthy development of both platform and restaurants can be promoted.

It is also found that higher promotion fees may require greater government regulation to ensure safe production by restaurants. By comparing situations (2) and (4), it can be seen that in situations where the platform’s overall revenues in situations (2) and (4) are similar and revenue from promotion fees accounts for a greater proportion, the government needs to impose more regulation (shifting from high to very high levels) to force restaurants to engage in safe production. The main reason is that restaurants are more sensitive to the promotion cost than the commission cost. Restaurants all exhibit varying degrees of loss aversion. However, for restaurants, there is a delayed and intermittent relationship between the promotion cost and the resulting profit. When a promotion cost is incurred, restaurants are more likely to perceive it as a loss rather than a general cost, which is similar to non-recoverable fees, which are recorded as sunk costs in accounting. In contrast, the commission cost is incurred along with the restaurants’ business operations. As long as the proportion of the commission cost does not result in a negative gross profit margin, restaurants are more likely to define it as a general operating cost rather than a loss. Hence, with the same total expenditure, the higher the proportion of promotion costs, the higher the loss aversion of restaurants. As a result, they are more inclined to risk illegal production to obtain higher profits to make up for the perceived loss, which means higher regulatory costs for the government to restrain restaurants from engaging in illegal production. Therefore, the government should encourage the platform to achieve profitability by charging lower promotion fees and higher commissions as far as possible.

## Validation of evolutionary game model based on case analysis

Meituan and Ele.me are the two most influential online food delivery platforms with the largest market shares in China, and received investment from Internet giants Tencent Group and Alibaba Group, respectively. The unique Internet technologies, strong financial strength, and valuable data resources of Tencent and Alibaba are the internal factors contributing to the monopoly of the two mentioned food delivery platforms. Thus, there are high entry barriers to establishing an online food delivery platform in China at this stage.

Founded in 2010, Meituan is an online food delivery platform receiving investment from an Internet giant. Over the years, with the increasing expansion of its capital and a continuous increase in consumer demand, Meituan has become one of the two titans of online food delivery in China. Its market share in online food delivery has increased each year and it now enjoys an absolute advantage (Table 8). Its share of the total transaction volume in China’s online food delivery market increased from 62.42% in 2017 to 75.17% in 2021, with an average annual increase of 3.17%.

**Table 8:** Online food delivery market size in China, and online food delivery transaction volume and market share of Meituan (100 million yuan, %).

Year	Online food delivery market size	Online food delivery transaction volume of Meituan	Market share of Meituan (%)
2017	2174	1711	62.42
2018	4250	2828	66.54
2019	5779	3927	67.95
2020	6646	4889	73.56
2021	9340	7021	75.17

However, with the platform’s rapid development, food safety in the restaurant industry has attracted widespread social attention in China. According to data disclosed by the China Consumers Association (2012–2021), the number of consumer complaints about the restaurant industry nationwide increased from 8595 in 2012 to 37,204 in 2021, an average annual growth rate of 36.98%. The significant increase in consumer awareness of their rights may be an important reason for this rapid increase. However, the annual growth rate of complaints in the restaurant industry was not only 22.14% higher than that about daily commodities in the same market environment during the same period, but also significantly higher than complaints about other commodities. For example, the number of complaints about medicines and medical supplies increased by an average of 9.41% annually from 2012 to 2021, and complaints about household electrical appliances even decreased by an average of 1.57%.

**Fig. 2:**
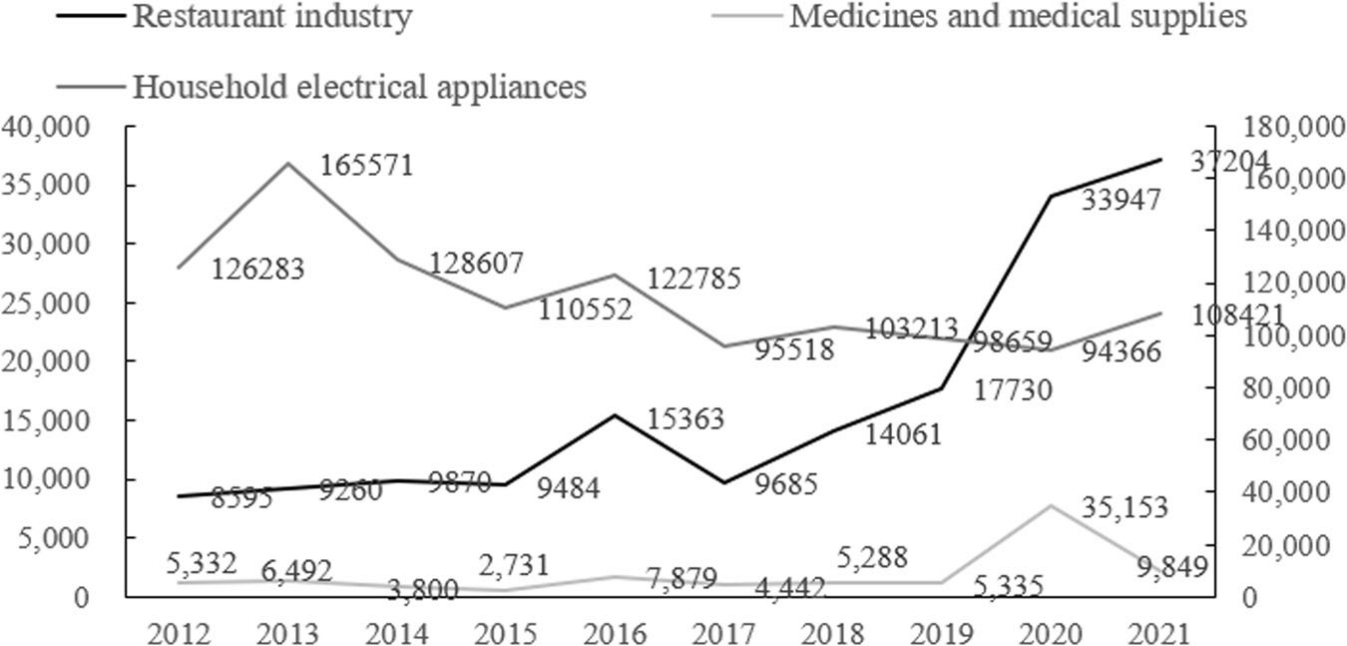
Complaint volumes about the restaurant industry, medicine and medical supplies, and household electrical appliances from 2012 to 2021 (*n*). It presents the complaint volumes about the restaurant industry, medicine and medical supplies, and household electronic appliances with different color lines from 2012 to 2021. The growth trends for complaints of different types were not the same.

**Fig. 3:**
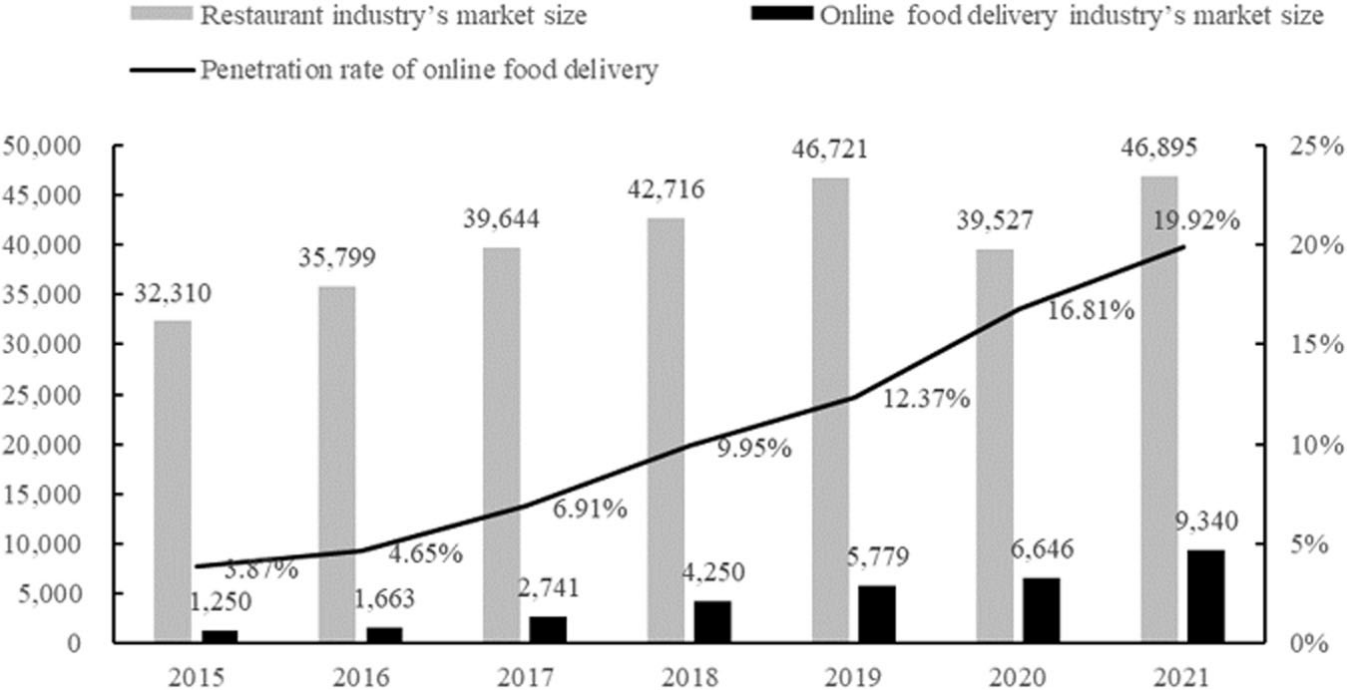
Market size of restaurant industry, and market size and penetration rate of online food delivery industry in China from 2015 to 2021 (100 million yuan, %). The gray and black columns show the restaurant industry’s market size and online food delivery industry’s market size, respectively. The line shows the penetration rate of online food delivery in China from 2015 to 2021. Source: National Bureau of Statistics of China. (2022). Annual data report of the restaurant industry. http://www.stats.gov.cn/ China Hotel Association, Ali New Service Research Center, Ele.me Training and Learning Center and Keruyun (2022). 2020–2021 Research Report on Food Delivery Industry Development in China. http://www.aliresearch.com/ch/information/informationdetails?articleCode=243631150608814080&type=%E6%96%B0%E9%97%BB.

Figure 2 presents the complaint volumes about the restaurant industry, medicine and medical supplies, and household electronic appliances from 2012 to 2021. It can be seen that although all these three types of commodities were affected by the COVID-19 pandemic in 2020 and 2021, the growth trends for complaints were not the same. Only the restaurant industry saw its complaint volume maintain rapid growth during the COVID-19 pandemic. The number of complaints about medicine and medical supplies, and household electronic appliances even decreased in 2021 and 2020, respectively. Considering the sharp increase in the penetration rate of online food delivery in China from 2015 to 2021, shown in Fig. 3, this paper speculates that the rapid and substantial increase in the number of complaints about the online food delivery industry is one of the main reasons for the high annual growth rate in the total complaint volume pertaining to the restaurant industry—and the root cause is problems with food quality and safety in online food delivery.

**Fig. 4:**
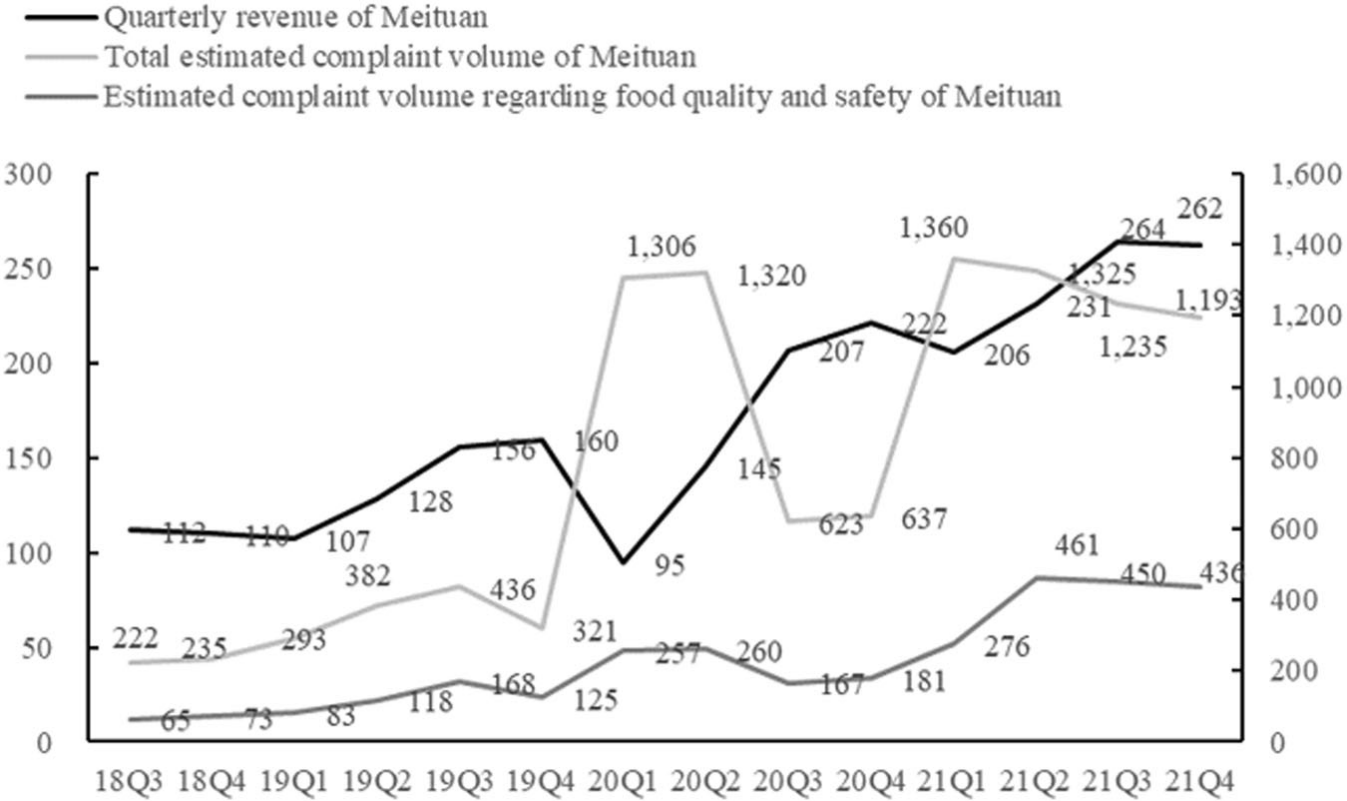
Quarterly operating income, total estimated complaint volume, and estimated complaint volume regarding food quality and safety of Meituan from 2018 to 2021 (100 million yuan, *n*). *Source*: Wind data service provider. Meituan-WHK3690 (2017–2020). Financial statements. http://stockpage.10jqka.com.cn/HK3690/China Consumers Association (2012–2021). Analysis of complaints received by consumers’ associations across China. https://www.cca.org.cn/.

According to (1) the penetration rate of online food delivery, (2) Meituan’s market share in the online food delivery market, and (3) the number of complaints about the entire restaurant industry, one can then estimate the number of complaints about Meituan regarding online food delivery (referred to as “estimated complaint volume of Meituan”). Similarly, the estimated complaint volume about Meituan regarding the quality and safety of online food delivery can also be calculated. Figure 4 shows the quarterly operating income, total estimated quarterly complaint volume, and estimated quarterly complaint volume regarding the food quality and safety of Meituan online food delivery from 2018 to 2021. The estimated complaint volume about Meituan increased from 222 in the third quarter of 2018 (18Q3 in Fig. 4) to 1193 in the fourth quarter of 2021 (21Q4 in Fig. 4). These changes in complaint volume showed the same growth pattern as the operating income of Meituan online food delivery and continued to increase. It indicates that the number of complaints increased with the increasing transaction volume of the online food delivery platform. Similarly, the complaint volume regarding food quality and safety also increased with increases in Meituan’s operating income, which initially indicates that the degree of capitalist profit-seeking is positively related to the food safety risks in online food delivery.

After its founding, Meituan saw its capital expansion go through an A round of investment in 2010, a B round of financing in 2011, a C round of financing in 2014, and a D round of financing in 2015, followed by substantial financing totaling US$3.3 billion in 2016, and listing on the Hong Kong Exchanges and Clearing Limited (HKEX) 2018. Receipt of such a large amount of capital has promoted the development of this online food delivery platform. Correspondingly, as shown in Fig. 4, the number of complaints caused by food quality and safety problems pertaining to the Meituan online food delivery platform has increased significantly. There may be many reasons for this increase. However, it cannot be overlooked that in the first quarter of 2020 (20Q1 in Fig. 4), which was during the outbreak of the COVID-19 pandemic in China, online food delivery became a necessity, and accordingly, industry associations and the vast majority of restaurants called for a reduction in platform commissions during this special period. However, the Meituan platform refused to reduce commissions. The profit-seeking nature of capital inherently required the Meituan online food delivery platform to prioritize creating short-term profits for shareholders over social responsibility.

Therefore, it can be argued that all capital-controlled online food delivery platforms, including Meituan, will inevitably take advantage of their ability to control the profitability of restaurants, impose blockades by virtue of their dominant market position, ensure their own economic interests by always pursuing high overall profits, and even compete for market profits by reaching exclusive dealing agreements that force an either-or choice.^4^ The monopolistic business model implemented by the Meituan online food delivery platform has greatly reduced the profit margins and even the operating environment that allows restaurants to stay afloat. As a result, restaurants are likely to engage in opportunistic behaviors, such as counterfeiting, price gouging, price dumping, and other illegal activities, which will inevitably increase the number of complaints due to food quality and safety problems in online food delivery.

The second significant surge in complaints about food quality and safety regarding the online food delivery platform occurred in the second quarter of 2021 (21Q2 in Fig. 4). The China Banking and Insurance Regulatory Commission requires banks to expand credit loans, continue to increase the number of first-time borrowers, promote loans that can be obtained and repaid at any time, and allocate more funds to small and micro enterprises and individual businesses. Due to this policy, the number of small and micro enterprises affiliated with the Meituan online food delivery platform increased significantly. However, no corresponding measures have been taken to guarantee food safety in online food delivery, and the government regulation strategy of online food delivery has not been adjusted accordingly, which has likely led to an increase in the number of complaints. Meanwhile, in 2021, the State Administration for Market Regulation of China publicly disclosed the illegal monopolistic practices of Meituan as a leading online food delivery company, which stimulated consumers’ awareness of their rights to a certain extent and affected the number of consumer complaints about Meituan. Therefore, changes in government policies and regulatory methods can affect the entire online food delivery industry.

## Research findings and policy implications

Based on the situation in China, this paper takes the Meituan online food delivery platform as an example to investigate the impact of capitalist profit-seeking by online food delivery platforms on food safety risks and to discuss the appropriate regulation level and its effectiveness. By analyzing the equilibrium situations generated by the platform’s profit strategies and the restaurants’ production strategies in the game, the following findings are established. First, capital-monopolized online platforms always pursue profit maximization. The platform’s profit-seeking strategies of high commissions or high promotion fees are very likely to reduce the living space of small, medium, and micro enterprises on the platform, forcing them to engage in illegal production to reduce costs, which greatly increases the food safety risks in online food delivery. This is consistent with the conclusion of Couldry and Mejias (2018) that large technology platforms may employ quantitative calculations to squeeze enterprises, as well as the findings of Li et al. (2020) that the measures implemented by restaurants to save costs also increase food safety risks. Second, the government needs to pay relatively high regulation costs to effectively compel restaurants to engage in safe production in order to reduce the food safety risks in online food delivery. However, although changes in government regulation levels can change the production strategies pursued by restaurants, they cannot change the platforms’ profit strategy. The platforms will not give up the pursuit of high overall profits, which fully verifies the profit-seeking nature of capital. Third and lastly, the higher the proportion of promotion fees in the profit strategy formulated by the platform, the more likely it is that the government will need to impose greater regulations to force restaurants to engage in safe production. The cost of government regulation can be reduced and efficiency improved by simply adjusting the proportions of commission and promotion fee charged by the platform without reducing the platform’s overall payoff or increasing the cost to consumers. Therefore, the findings of this study may provide new insights for the government to improve the regulation policy for food safety in online food delivery.

This study’s findings have important policy implications for the Chinese government. First, online food delivery platforms are essentially a product of the market economy. Therefore, the market should be allowed to play a decisive role, and hence the government should, first of all, respect the laws of the market economy. Second, online food delivery platforms provide directly edible food, which is a commodity and thus has attributes in common with all commodities. However, because restaurant food can directly impact the health of consumers, its public-goods attributes mean it has social responsibility higher than ordinary commodities. The monopolistic behavior resulting from the disorderly expansion of platform capital has threatened restaurant food safety. Therefore, the government should regulate the capital expansion of the platforms.

Third, the government’s role should be that of the regulator overseeing the operation of online food delivery platforms. One way it could increase the efficacy of its regulations is by implementing a negative list system that promotes fair competition in the restaurant market so as to maintain balance in the game of interests between the platform and restaurants and thus promote both parties to jointly engage in safe production to ensure food safety in online food delivery. Fourth, efforts should be made to improve the food safety risk management system used by online food delivery platforms. Meanwhile, the government should also encourage consumers to report food safety problems, support industry associations to participate in platform regulation, improve information transmission to reduce information asymmetry, and establish a social co-governance system. Fifth, the government should improve the market access mechanism for restaurants to access selling their products on these platforms and use the platforms’ own advanced information technology to reduce the cost of institutional regulation. Sixth and lastly, considering that the platforms hold a dominant market position in the game, the government can find a feasible way to improve regulatory efficiency without any reduction or only a slight reduction in the platform’s overall payoff and without increasing the cost to consumers.

Based on the online food delivery market data of China, this paper investigated the impact of capitalist profit-seeking by online food delivery platforms on food safety risks by constructing an evolutionary game model, and it has drawn meaningful conclusions. The model’s conclusions provide guidance for government regulation of online food delivery platforms in other countries. Future research can not only use data from other countries or online food delivery platforms for model testing and analysis but also focus on exploring other regulatory methods for online food delivery platforms to guide the healthy development of online food delivery transactions. For example, the current command-control system can be transformed into a laissez-faire regulatory system with scientific intervention to achieve consensus and balance between government intervention and market mechanisms. Moreover, a basic framework for coordinated regulation by the government, online platforms, and restaurants can be developed using the evolutionary game model to provide scientific decision-making support for ensuring food safety in online food delivery.

## Supplementary information

Data

## Data Availability

The datasets generated and analyzed during the current study are publicly available. The datasets are also available from the supplementary information in this paper.

## Footnotes

1 Penetration rate is the ratio of the target product or service to the product or service that the existing market may have, and is one of the methods to calculate the influence of a product or service. For example, Yeo and Kim (2019) assessed the global smartphone penetration rate.

2 Ghost kitchens, also known as cloud or dark kitchens, are restaurants that prepare meals solely for the delivery market, with no dine-in area or service staff (Li et al., 2020).

3 Opportunistic behavior refers to not fully or truthfully disclosing information in the case of information asymmetry and seeking self-interest at the expense of others, such as false advertising, patent infringement, violations of laws and regulations, and tax evasion (Williamson, 1985).

4 An either–or choice refers to the platform taking advantage of its dominant position and restaurants’ dependence on it to force restaurants to make an either-or choice between platforms, which is an abuse of market dominance and constitutes exclusive dealing.
